# High Antimalarial Efficacy of Dihydroartemisinin–Piperaquine on the China–Myanmar Border: The Calm before the Storm

**DOI:** 10.4269/ajtmh.15-0349

**Published:** 2015-09-02

**Authors:** Rick M. Fairhurst

**Affiliations:** Laboratory of Malaria and Vector Research, National Institute of Allergy and Infectious Diseases, National Institutes of Health, Rockville, Maryland

Dihydroartemisinin–piperaquine (DP) is the frontline artemisinin combination therapy (ACT) for uncomplicated falciparum malaria in several areas of southeast Asia, including Cambodia, Vietnam, and the China–Myanmar border. In this issue, Wang and others^1^ report that the clinical antimalarial efficacy of DP in northeastern Myanmar's Kachin State, near its border with China, was 100% in 2012–2013. Specifically, none of the 22 children or 49 adults who completed 42 days of follow-up developed recrudescent *Plasmodium falciparum* infections after treatment. Despite this finding, two potentially worrisome observations were made. First, 7% (5/71) of patients had detectable parasitemia on day 3, a reasonably good proxy for the presence of slow-clearing, artemisinin-resistant parasites. Second, 27% (19/71) of patients had gametocytemia, suggesting that their infections were transmissible before they sought treatment. Clearly, DP remains highly effective in clearing parasitemias in this area of Myanmar, but the regimen could benefit from the addition of primaquine to kill mature gametocytes and stop their transmission to others. This intervention is especially justified as 35% (38/109) of patients initially enrolled in this study were lost to follow-up because of family relocations.

It seems that not much has changed in Kachin State since 2008, when a similar study found that DP was 100% efficacious in 64 patients with falciparum malaria followed for 28 days and that 6% (4/64) of these patients had detectable parasitemia on day 3.^2^ But how well do these two studies forecast the current and future efficacy of DP in the surrounding region? Two relatively recent studies in neighboring Yunnan Province in China documented a slightly more concerning situation, reporting 100% efficacy and 18.5% day 3 positivity following artesunate monotherapy in 2009,^3^ and 96% efficacy and 13.6% day 3 positivity following DP therapy in 2010.^4^ Although these studies suggest that DP remained highly effective along the China–Myanmar border in 2008–2010, the day 3 positivity rates exceeding 10% in Yunnan Province suggest that artemisinin-resistant parasites have been circulating in this border region for the past 6 years. Unfortunately, this possibility was recently confirmed by Huang and others,^5^ who found that nearly half of all infections sampled in Yunnan Province near the Myanmar border in 2009–2012 carried *kelch13* propeller domain mutations—a genetic marker for in vitro and in vivo artemisinin resistance in southeast Asia.^6^,^7^ One particular mutation, F446I, predominated in this study^5^ with a prevalence of 36.5%, and was associated with delayed parasite clearance—the clinical hallmark of artemisinin resistance.

If past is prologue, this level of artemisinin resistance will cause DP failures to emerge rapidly along the China–Myanmar border. This is because the excess parasites that survive artemisinin must be eliminated by piperaquine alone, a pharmacodynamics–pharmacokinetics setting in which parasites are more likely to acquire genetic resistance to piperaquine and thereby survive DP. This phenomenon appears to have evolved quickly in western Cambodia, the epicenter of artemisinin resistance, in 2009–2013 when DP was being widely used and *kelch13* mutations were rapidly spreading. In 2008–2010, for example, the clinical efficacy of DP in two western Cambodian provinces (Pailin and Pursat) was 75% and 89%, respectively, but it was 100% in eastern Cambodia, where artemisinin resistance is still very uncommon.^8^ More recently in 2013, the clinical efficacy of DP in another western Cambodian province (Oddar Meanchey) was even lower, at 46%.^9^ Together, these data indicate that parasites have rapidly become resistant to DP along the Cambodia–Thailand border, where the clinical efficacy of DP was 96–98% as recently as 2001–2005. Since DP has been used along the China–Myanmar border since 2009, and the prevalence of *kelch13* mutations is high in Yunnan Province^5^ and increasing in Kachin State,^1^ the writing is on the wall. DP failures will emerge soon and spread efficiently, especially given current patterns of human migration and high rates of untreated gametocytemia in the region.

Obviously, now is the time to plan for the inevitability of increasing DP resistance in Myanmar. Given that therapeutic efficacy studies are expensive, logistically challenging, and focused on limited geographic areas, the need for a comprehensive, collaborative, and cost-reducing approach to real-time surveillance for *kelch13* mutations throughout southeast Asia is urgent. In areas such as Yunnan Province where the prevalence of *kelch13* mutations is already high,^5^ the top research priorities should be the continuous monitoring of DP efficacy (especially since a marker of piperaquine resistance is presently lacking) and the early testing of alternative ACTs. Since artesunate plus mefloquine, artemether–lumefantrine, and artesunate–pyronaridine may be suitable replacements for DP, the next round of clinical studies will likely compare the efficacy of one or more of these ACTs to that of DP. Although parasite resistance to lumefantrine has not yet been described and pyronaridine has not yet been widely used, ACTs containing these partner drugs may not be as effective in areas where artemisinin resistance is common. Measuring the ex vivo susceptibility of parasites to mefloquine and the prevalence of multiple *pfmdr1* copies—a genetic marker for mefloquine resistance—may be helpful in determining whether artesunate plus mefloquine can treat DP failures or replace DP as a frontline ACT in some areas.^10^ Indeed, such phenotypic and genotypic data recently supported the reinstatement of artesunate plus mefloquine as the frontline ACT in five western Cambodian provinces where DP failures are unacceptably high.

In the meantime, measures that may forestall the emergence and spread of DP failures along the China–Myanmar border include vector avoidance and control measures, proper dosing of DP in children (who clear piperaquine more rapidly than adults), and the use of primaquine to prevent transmission. Although single low-dose primaquine is believed to kill stage-V gametocytes, the mosquito-transmissible form of *Plasmodium falciparum*, the effect of this drug on the gametocytes of artemisinin-resistant parasites has not been established. The recent association between slow-clearing *kelch13* mutants and increased development of gametocytemia following artesunate plus DP in western Cambodia^7^ suggests that *kelch13* mutations confer artemisinin resistance to immature gametocytes. This hypothesis, as well as the possibility that *kelch13* mutations also confer some level of resistance to primaquine—a prooxidant drug like artemisinin—warrants immediate investigation. As artemisinin resistance further entrenches itself in southeast Asia, the adverse impacts of untreated gametocytemia and human migration on malaria control will likely worsen in this region and thereby increase the threat level for Africa, where malaria's greatest transmission, morbidity, and mortality are concentrated.

## References

[1] Wang Y, Yang Z, Yuan L, Zhou G, Parker D, Lee MC, Yan G, Fan Q, Xiao Y, Cao Y, Cui L (2015). Clinical efficacy of dihydroartemisinin-piperaquine for the treatment of uncomplicated *Plasmodium falciparum* malaria at the China-Myanmar border. Am J Trop Med Hyg.

[2] Sun XD, Zhang ZX, Wang J, Deng Y, Yang YC, Lasi JH, Sun XY, Wang H (2011). Therapeutic efficacy and safety of compound dihydroartemisinin/piperaquine for uncomplicated *Plasmodium falciparum* infection in Laiza City of Myanmar bordering on China. Zhongguo Ji Sheng Chong Xue Yu Ji Sheng Chong Bing Za Zhi.

[3] Huang F, Tang L, Yang H, Zhou S, Sun X, Liu H (2012). Therapeutic efficacy of artesunate in the treatment of uncomplicated *Plasmodium falciparum* malaria and anti-malarial, drug-resistance marker polymorphisms in populations near the China-Myanmar border. Malar J.

[4] Bustos MD, Wongsrichanalai C, Delacollette C, Burkholder B (2013). Monitoring antimalarial drug efficacy in the Greater Mekong Subregion: an overview of in vivo results from 2008 to 2010. The Southeast Asian J Trop Med Public Health.

[5] Huang F, Takala-Harrison S, Jacob CG, Liu H, Sun X, Yang H, Nyunt MM, Adams M, Zhou S, Xia Z, Ringwald P, Bustos MD, Tang L, Plowe CV (2015). A single mutation in K13 predominates in southern China and is associated with delayed clearance of Plasmodium falciparum following artemisinin treatment. J Infect Dis.

[6] Ariey F, Witkowski B, Amaratunga C, Beghain J, Langlois AC, Khim N, Kim S, Duru V, Bouchier C, Ma L, Lim P, Leang R, Duong S, Sreng S, Suon S, Chuor CM, Bout DM, Ménard S, Rogers WO, Genton B, Fandeur T, Miotto O, Ringwald P, Le Bras J, Berry A, Barale JC, Fairhurst RM, Benoit-Vical F, Mercereau-Puijalon O, Ménard D (2014). A molecular marker of artemisinin-resistant Plasmodium falciparum malaria. Nature.

[7] Ashley EA, Dhorda M, Fairhurst RM, Amaratunga C, Lim P, Suon S, Sreng S, Anderson JM, Mao S, Sam B, Sopha C, Chuor CM, Nguon C, Sovannaroth S, Pukrittayakamee S, Jittamala P, Chotivanich K, Chutasmit K, Suchatsoonthorn C, Runcharoen R, Hien TT, Thuy-Nhien NT, Thanh NV, Phu NH, Htut Y, Han KT, Aye KH, Mokuolu OA, Olaosebikan RR, Folaranmi OO, Mayxay M, Khanthavong M, Hongvanthong B, Newton PN, Onyamboko MA, Fanello CI, Tshefu AK, Mishra N, Valecha N, Phyo AP, Nosten F, Yi P, Tripura R, Borrmann S, Bashraheil M, Peshu J, Faiz MA, Ghose A, Hossain MA, Samad R, Rahman MR, Hasan MM, Islam A, Miotto O, Amato R, MacInnis B, Stalker J, Kwiatkowski DP, Bozdech Z, Jeeyapant A, Cheah PY, Sakulthaew T, Chalk J, Intharabut B, Silamut K, Lee SJ, Vihokhern B, Kunasol C, Imwong M, Tarning J, Taylor WJ, Yeung S, Woodrow CJ, Flegg JA, Das D, Smith J, Venkatesan M, Plowe CV, Stepniewska K, Guerin PJ, Dondorp AM, Day NP, White NJ, Tracking Resistance to Artemisinin Collaboration (TRAC) (2014). Spread of artemisinin resistance in *Plasmodium falciparum* malaria. N Engl J Med.

[8] Leang R, Barrette A, Bouth DM, Menard D, Abdur R, Duong S, Ringwald P (2013). Efficacy of dihydroartemisinin-piperaquine for treatment of uncomplicated *Plasmodium falciparum* and *Plasmodium vivax* in Cambodia, 2008 to 2010. Antimicrob Agents Chemother.

[9] Spring MD, Lin JT, Manning JE, Vanachayangkul P, Somethy S, Bun R, Se Y, Chann S, Ittiverakul M, Sia-Ngam P, Kuntawunginn W, Arsanok M, Buathong N, Chaorattanakawee S, Gosi P, Ta-Aksorn W, Chanarat N, Sundrakes S, Kong N, Heng TK, Nou S, Teja-Isavadharm P, Pichyangkul S, Phann ST, Balasubramanian S, Juliano JJ, Meshnick SR, Chour CM, Prom S, Lanteri CA, Lon C, Saunders DL (2015). Dihydroartemisinin-piperaquine failure associated with a triple mutant including kelch13 C580Y in Cambodia: an observational cohort study. Lancet Infect Dis.

[10] Lim P, Dek D, Try V, Sreng S, Suon S, Fairhurst RM (2015). Decreasing pfmdr1 copy number suggests that *Plasmodium falciparum* in western Cambodia is regaining in vitro susceptibility to mefloquine. Antimicrob Agents Chemother.

